# Acute Esophageal Mucosal Lesion Mimicking Severe Reflux Esophagitis in Diabetic Ketoacidosis: A Diagnostic Pitfall

**DOI:** 10.3390/diagnostics16101566

**Published:** 2026-05-21

**Authors:** Yohei Midori, Koji Hayashi, Maho Hayashi, Hidetaka Matsuda

**Affiliations:** 1 Department of Gastroenterology, Fukui General Hospital, 28-16-1, Egami-Cho, Fukui 910-8561, Japan; hidem@u-fukui.ac.jp; 2 Department of Rehabilitation Medicine, Fukui General Hospital, 28-16-1, Egami-Cho, Fukui 910-8561, Japan; 3 Department of Neurology, University of Fukui, 23-3 Matsuoka-Shimoaizuki, Eiheiji-Cho, Yoshida-Gun, Fukui 910-1193, Japan; 4 Department of Internal Medicine, Fukui General Hospital, 28-16-1, Egami-Cho, Fukui 910-8561, Japan

**Keywords:** acute esophageal mucosal lesion, acute esophageal necrosis, black esophagus, diabetic ketoacidosis, esophagitis, gastroesophageal reflux, gastroscopy

## Abstract

A 65-year-old man with type 2 diabetes presented with abdominal pain. Although he had no typical reflux symptoms such as heartburn or acid regurgitation, esophagogastroduodenoscopy (EGD) showed findings suggestive of reflux esophagitis, and proton pump inhibitor therapy was initiated. Two months later, he was admitted with intractable vomiting. EGD demonstrated diffuse circumferential mucosal injury without black discoloration, predominantly in the distal esophagus. These findings were interpreted as severe reflux esophagitis (Los Angeles grade D; RE-D). Symptoms improved with supportive care, glycemic control, and continued PPI therapy; follow-up EGD showed marked improvement. Six months later, he re-presented with identical symptoms and endoscopic findings. Laboratory testing confirmed diabetic ketoacidosis (DKA), with ketonuria, elevated total ketone bodies (2469 µmol/L), and high-anion gap metabolic acidosis (anion gap 17.2 mEq/L). The diagnosis was revised to DKA-associated acute esophageal mucosal lesion (AEML). He improved with fluid resuscitation and insulin therapy, and medication adherence was reinforced. Follow-up EGD showed complete healing without recurrence. AEML has been proposed as a spectrum that includes acute esophageal necrosis (AEN; “black esophagus”) and esophagitis without black-appearing mucosa. This case highlights a diagnostic pitfall in which DKA-associated AEML without black discoloration may be misattributed to severe reflux esophagitis. When the clinical presentation or endoscopic appearance is severe or atypical, clinicians should consider AEML and evaluate for underlying systemic precipitants.

**Figure 1:**
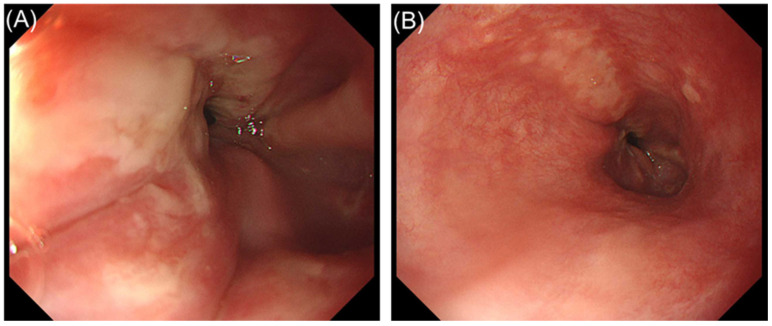
Endoscopic findings at initial presentation. (**A**) Mucosal breaks covered with whitish exudate at the esophagogastric junction that extend between the tops of two or more mucosal folds. (**B**) Multiple longitudinal mucosal breaks with whitish exudate in the distal esophagus. These findings were interpreted as reflux esophagitis (Los Angeles grade C) [1].

**Figure 2:**
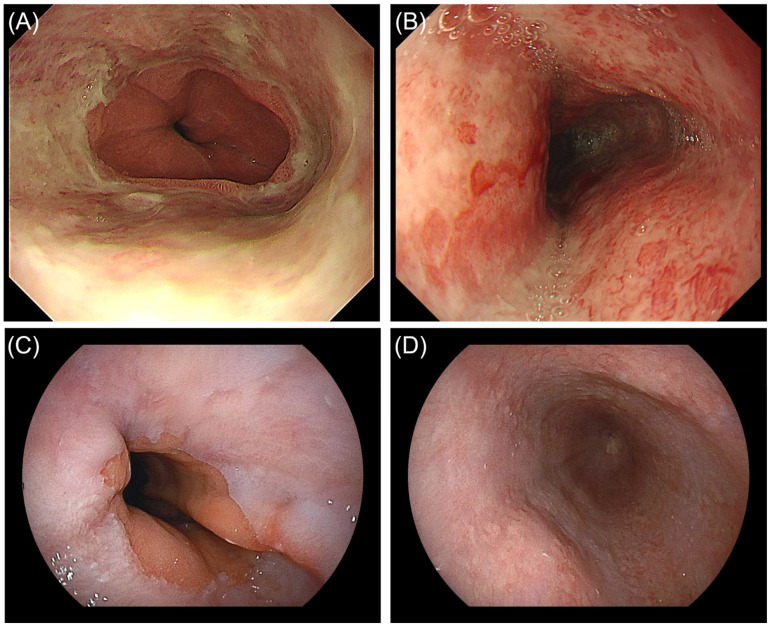
Endoscopic findings two months after the initial presentation with intractable vomiting and at follow-up esophagogastroduodenoscopy (EGD). (**A**) Esophagogastric junction (EGJ), showing circumferential mucosal injury with an abrupt cutoff at the EGJ and no black discoloration. (**B**) Mid-esophagus, showing circumferential mucosal injury with erythema. These findings were interpreted as severe reflux esophagitis (Los Angeles grade D) [1]. Follow-up EGD 1 month after discharge showed improvement of the mucosal injury at the EGJ (**C**) and mid-esophagus (**D**).

**Figure 3:**
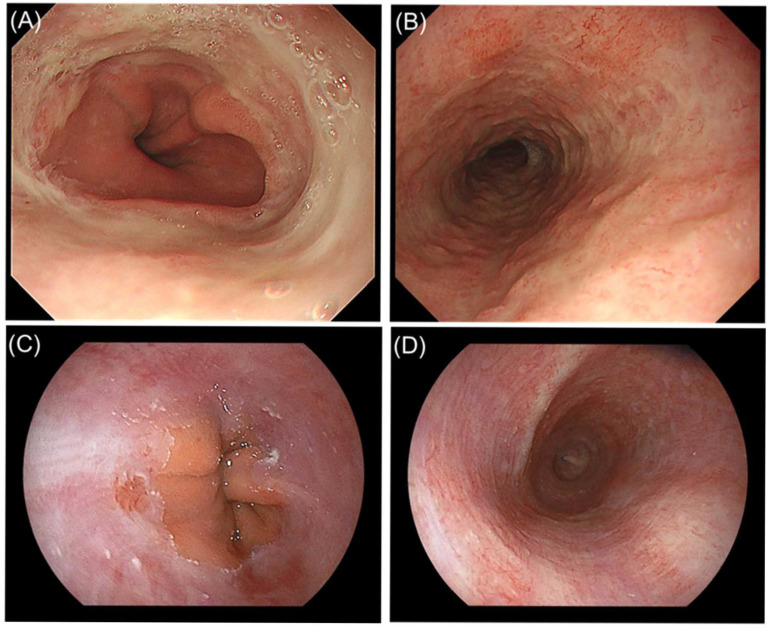
Endoscopic findings at recurrence six months later, when the diagnosis was revised to DKA-associated AEML, and at follow-up esophagogastroduodenoscopy (EGD). (**A**) Esophagogastric junction (EGJ), showing circumferential mucosal injury with an abrupt cutoff at the EGJ and no black discoloration, similar to the findings six months earlier. (**B**) Mid-esophagus, showing circumferential mucosal injury without clear radial tapering. (**C**,**D**) Follow-up EGD at 1 month demonstrated mucosal improvement at the EGJ and mid-esophagus, with partial scarring in the mid-esophagus. AEML is a proposed disease concept encompassing acute esophageal necrosis (AEN; “black esophagus”) and esophagitis without black-appearing mucosa [2], and can develop secondary to systemic diseases, including DKA [3,4]. AEN is considered the severe form of AEML [5] and typically presents as circumferential black distal esophageal mucosa [4]. Non-black esophagitis within the AEML spectrum lacks black discoloration and can closely resemble severe reflux esophagitis (Los Angeles grade D; RE-D) on endoscopy, creating a diagnostic pitfall. Although differences in the proximal extent (untapered circumferential extension vs. radial tapering) have been proposed as endoscopic clues to distinguish AEML from RE-D, appearance alone may be insufficient for reliable differentiation; therefore, evaluation of underlying systemic disease is essential [6]. In the present case, the patient did not report typical reflux symptoms such as heartburn or acid regurgitation at the initial presentation or during either subsequent admission with vomiting. In this context, the discrepancy between the atypical symptom profile and the severe reflux-esophagitis-like endoscopic appearance may be helpful in prompting consideration of AEML and underlying systemic disease. Among systemic precipitants, DKA has been reported as the most frequent precipitating condition for AEML in some case series [7]. The mechanism of DKA-associated AEML remains unclear but is likely multifactorial. Osmotic diuresis and fluid loss in DKA may lead to hypovolemia and esophageal mucosal hypoperfusion, while vomiting, gastric fluid stasis, and gastroesophageal reflux may increase chemical injury from gastric contents. These ischemic and chemical factors may together contribute to acute esophageal mucosal injury [8]. However, many published reports in the setting of DKA describe AEN presenting as black esophagus in severe DKA [8,9], whereas AEML without black discoloration has been described less frequently. DKA can present with gastrointestinal symptoms [10] and should be considered in the differential diagnosis, particularly to avoid misdiagnosis as RE-D, as in the present case. This case highlights a diagnostic pitfall in which DKA-associated AEML without black discoloration may be misattributed to severe reflux esophagitis. Clinicians should consider AEML and evaluate for underlying systemic precipitants when the appearance is severe or atypical, rather than relying on endoscopic appearance alone.

## Data Availability

The data presented in this study is available on request from the corresponding author. Due to patient privacy and ethical considerations, the data is not publicly accessible.

## Abbreviations

The following abbreviations are used in this manuscript:

EGD	Esophagogastroduodenoscopy
RE-D	Severe reflux esophagitis (Los Angeles grade D)
DKA	Diabetic ketoacidosis
AEML	Acute esophageal mucosal lesion
EGJ	Esophagogastric junction
AEN	Acute esophageal necrosis
